# Insecticide-treated net ownership, utilization and knowledge of malaria in children residing in Batoke–Limbe, Mount Cameroon area: effect on malariometric and haematological indices

**DOI:** 10.1186/s12936-021-03860-6

**Published:** 2021-07-29

**Authors:** Rene Ning Teh, Irene Ule Ngole Sumbele, Derick Ndelle Meduke, Gillian Asoba Nkeudem, Samuel Takang Ojong, Exodus Akwa Teh, Helen Kuokuo Kimbi

**Affiliations:** 1 Department of Zoology and Animal Physiology, University of Buea, Buea, Cameroon; 2 Department of Social Economy and Family Management, Higher Technical Teachers’ Training College, University of Buea, Kumba, Cameroon; 3 Clinical Diagnostic Laboratory, University of Buea, Buea, Cameroon; 4 Department of Microbiology, Kenyatta University, Nairobi, Kenya; 5 Department of Medical Laboratory Sciences, The University of Bamenda, Bambili, Cameroon; 6 Department of Microbiology and Immunology, Cornell College of Veterinary Medicine, Ithaca, NY USA

**Keywords:** Malaria, ITN, Ownership, Utilization, Anaemia, Knowledge, Children

## Abstract

**Background:**

Insecticide-treated nets (ITNs) are the most widely used interventions for malaria control in Africa. The aim of this study was to assess the ownership and utilization of ITNs and the knowledge of malaria and their effects on malariometric and haematological indices in children living in the Mount Cameroon area.

**Methods:**

A community-based cross-sectional study involving a total of 405 children aged between 6 months and 14 years living in Batoke–Limbe was carried out between July and October 2017. A semi-structured questionnaire was used to document demographic status, knowledge on malaria and ITN ownership and usage. Venous blood sample was collected from each child to determine the prevalence and intensity of parasitaemia by Giemsa-stained microscopy and full blood count by auto haematology analysis to obtain white blood cell (WBC) and red blood cell (RBC) counts, haemoglobin (Hb) level, haematocrit (Hct), mean corpuscular volume (MCV), mean corpuscular haemoglobin (MCH) and mean corpuscular haemoglobin concentration (MCHC). A multilinear regression model was used to determine the relationship between haematological parameter as dependent variable and the independent variables.

**Results:**

The overall prevalence of parasitaemia, anaemia, knowledge about malaria, ITN ownership, usage and effective usage was 46.7%, 54.7%, 40.7%, 78.8%, 50.9% and 29.9%, respectively. The prevalence of parasitaemia was significantly higher (P < 0.001) in children who ineffectively utilized ITNs (54.9%) than effective users (27.3%). Having knowledge of malaria, negatively correlated with WBC counts (P = 0.005), but positively correlated with Hb levels (P < 0.001), RBC counts (P < 0.001), Hct (P < 0.001), MCV (P < 0.001) and MCH (P < 0.001). ITN use positively correlated with WBC counts (P = 0.005) but negatively with Hb levels (P = 0.004), RBC counts (P = 0.006), and MCH (P < 0.001). Meanwhile, parasitaemia negatively correlated with Hb levels (P = 0.004), RBC counts (P = 0.01), Hct (P = 0.04) and MCHC (P = 0.015).

**Conclusion:**

There is need for more sensitization on the benefits of using the ITNs to meet up with the intended and expected impact of the free distribution of ITNs.

**Supplementary Information:**

The online version contains supplementary material available at 10.1186/s12936-021-03860-6.

## Background

Globally, malaria is still a public health concern as 228 million cases of malaria occurred worldwide in 2018 compared with 251 million cases in 2010 and 231 million cases in 2017, with Cameroon accounting for 3% of the total number [1]. Also, an estimated 445,000 deaths was caused by malaria in 2016 and Cameroon alone accounted for 3% of this number [2], despite the control measures put in place [3–5]. Insecticide-treated nets (ITNs) are effective tools for malaria prevention and have been shown to significantly reduce malaria episodes, severe disease, and malaria-related deaths especially among children aged less than five years in endemic areas [6]. At high coverage levels, ITNs provide both individual and community protection for both users and non-users by killing the *Anopheles* vector, thereby considerably reducing their longevity and entomological inoculation rate [7]. Lengeler [8] reported that ITNs had helped to reduce malaria episodes by 48–50% and in addition, Bhatt et al*.* [9], reported that between 2001 and 2015, malaria parasite prevalence in endemic countries reduced by 50%, with 68% of this decline attributed to the use of ITNs. Conversely, some studies have reported an increase in malaria parasites after increasing the coverage of ITN [10–12].

In 2018, the World Health Organization (WHO) [1], reported that 50% of the people at risk of malaria in sub-Saharan Africa slept under an ITN, with half of the population being protected by this intervention, an increase from 29% in 2010. In order to achieve the Abuja target of 80% usage and reduce malaria burden in Cameroon, so far, progress has been made in the distribution of long-lasting insecticidal nets (LLINs), particularly through campaigns for more than 8 million to 12 million LLINs were distributed between 2011 and 2016 [13]. Yet, proportions (18.2%) of these nets are used for other purposes, such as fishing, nursing seeds and football nets [6]. In some cases, they are not used at all for varied reasons such as heat, the feeling of being in a coffin, dislike of colour or pregnant woman keeping it to use for the new-born. So, ownership does not necessarily translate to utilization. Therefore, this could undermine the aim of the 2016 net distribution campaign. Studies carried out in the Mount Cameroon area reported a higher proportion of households with at least one ITN (77.6%), with a low bed space coverage of 58.5% [14], indicating that coverage defined by the WHO as one ITN for every two persons remains a challenge. Furthermore, several intervention studies have been carried out on malaria and some enhanced control measures [4, 5, 15] in the Mount Cameroon area, and the findings revealed ITN efficacy in reducing malaria parasite infection, although such drop may not be homogenous across the country. However, there are also concerns on the increasing pyrethroid resistance, which is likely to affect LLIN efficacy in preventing malaria parasite infection. Even though a study in Cameroon [16] as well as various studies in Malawi [17], Benin [18, 19] and several countries in Africa [20] reported that LLINs still offers some protection even in areas of high pyrethroid resistance, there is need for continuous surveillance and evaluation on the effective use of LLINs distributed by the Cameroon government and its partners to reduce malaria mortality and morbidity.

Irrespective of the increased ownership of ITNs/LLINs, a decrease in malaria transmission or morbidity is still to be appreciated especially as Cameroon is reported to harbour the five most efficient and common malaria vectors (*Anopheles gambiae, Anopheles arabiensis, Anopheles funestus, Anopheles nili* and *Anopheles moucheti*) [21]. Moreover, the effective control of malaria within a community is affected by their cultural beliefs [22, 23]. In addition, Tyagi et al. [23] reported that community knowledge about malaria causation, symptoms, treatment and prevention has been linked to the inability of malaria programs to achieve sustainable control. It is thus imperative to evaluate the influence of the ownership and utilization of treated mosquito bed nets, malaria knowledge and it influence on the malariometric and haematological indices in children living in a malaria endemic zone, such as Batoke–Limbe, in the Mount Cameroon area.

## Methods

### Study area and participants

The study was conducted in Batoke–Limbe a coastal community in the Fako Division of the South West Region of Cameroon. The area is subjected to a Cameroonian-type equatorial climate characterized by fairly constant temperatures and two seasons. Batoke–Limbe is situated at the foot of Mount Cameroon and is bounded to the west by the Atlantic Ocean. The area has been described in detailed by Teh et al*.* [24].

**Fig. 1:**
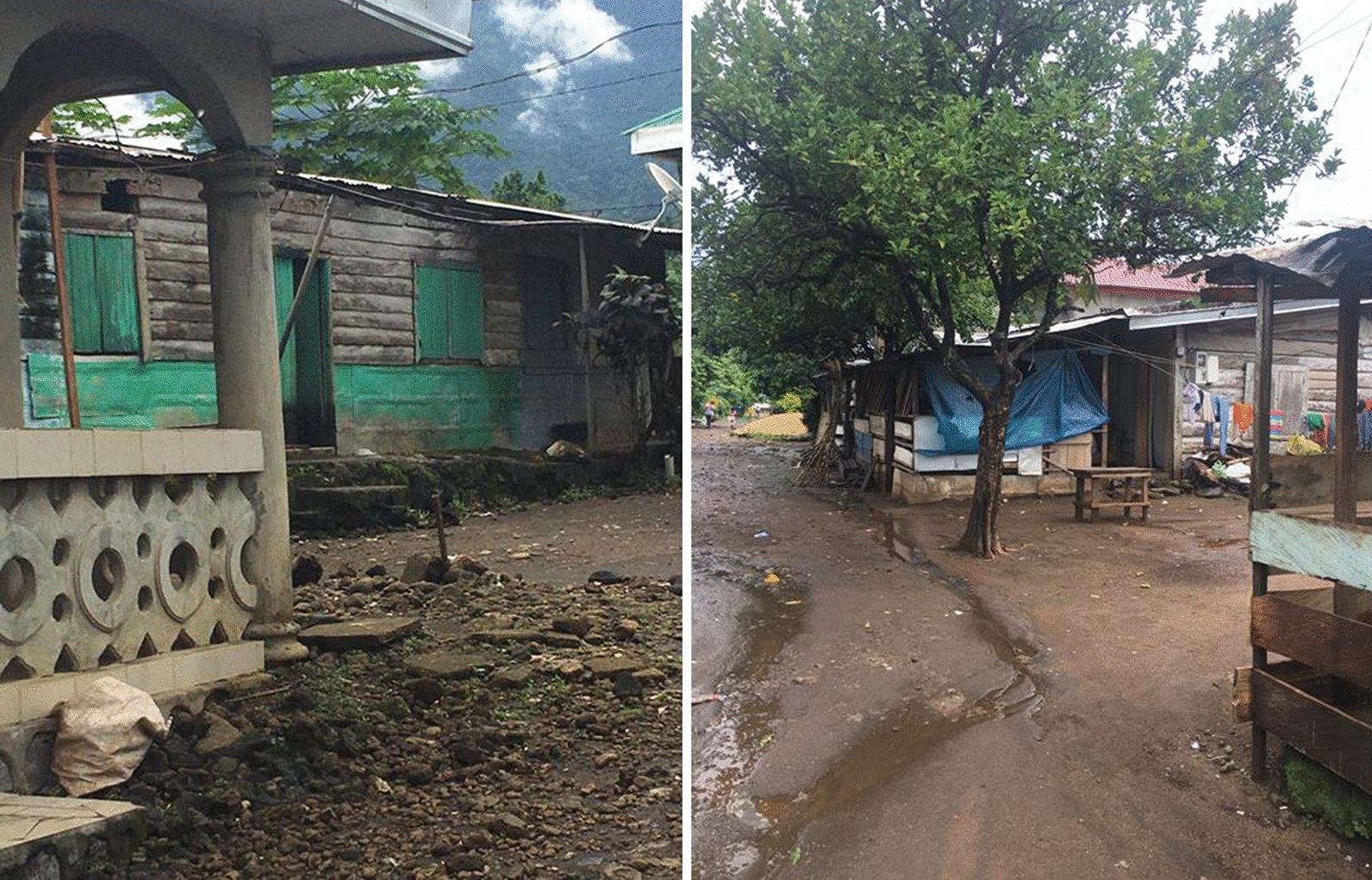
Plank houses with crevices on the walls

Fishing, oil palm plantation agriculture and small-scale peasant farming are the main agricultural practices in this community. The main house type is mostly plank, although block houses are also common. Some of the plank houses are quite old with many crevices on the walls and many others have no ceiling or a window mosquito mesh as seen in Fig. 1.

All children ≤ 14 years in Batoke–Limbe whose parents/caregivers signed the consent/assent forms and had resided in the area for at least a month, were recruited in the study. Children who presented with high fever or any other medical conditions requiring intensive care hospitalization, were excluded from the study.

### Study design

The community-based cross-sectional study was conducted between the months of July to October 2017, during the peak malaria transmission period in the Mount Cameroon area [25]. Participants were invited to the data collection location in each community by their local chiefs and coordination was organised by the head/leader of a block within a neighbourhood (quarter head) of the various communities. Following prior sensitization of the community, a date for collection of samples was made for each family to ensure the participation of at least a child less than or equal to 14 years in the study.

### Sample size and population

The sample size was calculated using the following formula [26]:

$${\text{n}} = {\text{Z}}^{2} {\text{pq}}/{\text{d}}^{2} ;$$

where z = 1.96: confidence level test statistics at the desired level of significance, q = 1 − p: proportion of malaria parasite negative and d = acceptable error willing to be committed, P = 29.6%, prevalence of malaria in children ≤ 14 years in the study area [15]. The minimum estimated sample size calculated was approximately 325. After adding 15% non-response rate, the sample size obtained was 382. The required sample size was attained after a convenient sampling method was used in all the blocks. The final sample size was adjusted to a minimum of 400 participants to account for anticipated incomplete data entry and blood loss by clotting. The study participants included pairs of children between 6 months and 14 years and their parents or caregiver.

### Data collection

Semi-structured questionnaires, with questions on ITN usage and its state, derived from Sumbele et al. [4] were administered to the parents/guardians/caregivers. Pidgin, English or French languages were used to administer the questionnaire depending on the language of choice requested by the respondent. ITN use was defined as having slept under an ITN the night prior to the survey. Children, who had good nets, used them regularly and slept before 10 pm, were considered as effective users. All reported ITNs were inspected except in some cases where inspection was not possible (locked room). The physical integrity of each ITN was assessed by checking for holes in the nets and counting them using the WHO Pesticide Evaluation Scheme (WHOPES), which defined sizes as recommended by the WHO [27].

Relating to the knowledge and scoring of malaria; the questions relevant to knowledge of malaria in the questionnaire were derived from Kimbi et al*.* [28]. For analysis, a total of 13 items were included in the knowledge section which included signs and symptoms, effects, transmission and control measures. The combined level of knowledge was classified according to each respondent’s score. Appropriate knowledge corresponded to a score greater than the mean score while inappropriate knowledge corresponded to a score less than the mean.

The axillary body temperature of each child was measured using an electronic thermometer and fever was classified as body temperature ≥ 37.5 °C.

### Malaria parasite detection and assessment

Out of the 4 ml of blood collected by venepuncture using a sterile 5 ml syringe, 6 and 3 µl were dispensed immediately on the same slide for the preparation of thick and thin blood films, respectively. The films were Giemsa-stained and examined in the laboratory following standard procedures [29]. The malaria parasite density was determined from the thick blood film by counting the number of parasites per 200 leukocytes on thick blood film and multiplying the parasite count with the participants’ white blood cell count obtained from the complete blood count analysis. Malaria parasite density was categorized as low (< 1000 parasites/μl blood), moderate (1000–4999 parasites/μl blood), high (5000–99,999 parasites/μl blood), and hyper parasitaemia (≥ 100,000 parasites/μl blood) [4].

### Determination of haematological parameters

Briefly, the blood samples were placed on a multi mixer rotator for uniform mixing. A complete blood count was run following the manufacturer’s instructions using an auto-haematology analyser (MINRAY 2800 BC) to obtain haemoglobin concentration (Hb), haematocrit (Hct), mean corpuscular volume (MCV), mean corpuscular haemoglobin concentration (MCHC), mean corpuscular haemoglobin (MCH), and red blood cell distribution width coefficient of variation (RDW-CV). Anaemia was defined as Hb < 11 g/dl and further classified as mild (Hb, 10.1–10.9 g/dl), moderate (Hb, 7.0–10.0 g/dl) and severe (Hb < 7 g/dl) [30].

### Statistical analysis

Data was entered into log books and later entered into spread sheets using Microsoft Excel. After data cleansing, analysis was performed using the Statistical Package for Social Sciences (SPSS) version 20 (IBM-SPSS, Inc, Chicago, IL, USA) and Epi–Info version 7 software. Data was summarized into means and standard deviations (SD), and percentages were used in the evaluation of the descriptive statistics. The comparison between malaria parasite, anaemia, ITN (ownership and usage), as dependent variables and demographic and clinical variables as independent variables were evaluated using the Chi-square test (χ^2^). Malaria parasite counts were log transformed before analysis. The geometric mean parasite densities (GMPDs) were used to compare the intensity of infection in the study population and differences were compared using Mann–Whitney U test and Kruskal–Wallis test where appropriate. The combined level of knowledge of malaria was classified according to each respondent’s score. The mean knowledge score was 5. Appropriate knowledge corresponded to a score greater than or equal to 5, while inappropriate knowledge corresponded to a score less than 5. A multilinear regression model was used to determine the relationship of each haematological parameter as dependent variable and age, gender, knowledge about malaria, malaria parasite status, ITN use, and effective use of ITN as independent variables. Significant levels were measured at 95% confidence interval (CI) with significant differences designated at P < 0.05.

### Ethical considerations and administrative approval

Ethical approval for the study was obtained from the Institutional Review Board of the Faculty of Health Sciences, University of Buea (2017/004/UB/FHS/IRB) following an administrative clearance from the South West Regional Delegation of Public Health, Cameroon. Informed consent/assent forms were read, given and explained to parents or caregivers of the children at presentation. The role of the participants was well explained and only participants who gave written and/or verbal consent or assent took part in the study. Even though participation was voluntary, parents or caregivers were free at any point to terminate the participation of the child/children in the study.

## Results

### Characteristics of the study population

A total of 405 children with a mean (SD) age of 6.31(3.54) years, and of both sexes; males (48.9%, 198) and females (51.1%, 207) residing in Batoke–Limbe participated in the study. The ITN ownership (N) was 78.8% (319) with a utilization of 50.9% (206). Most of the parents/ guardians of the children had a primary level of education (45.9%, 170) with a few (5.1%, 19) having no formal education. The proportion of children who effectively used a mosquito net was 29.9% (121), with a great proportion of the nets in good condition (Table 1). The proportion of fever, MP and anaemia in the study population were 7.9% (32), 46.7% (189) and 54.6% (221), respectively as shown in Table 1.

**Table 1:** Socio-demographic and clinical characteristics of the study population

Parameter	% (N)
Number of participants	100 (405)
Mean age (SD) in years	6.31 (3.54)
Age groups (years)	
< 5	37.8 (153)
5–9	40.2 (163)
10–14	22 (89)
Sex	
Male	48.9 (198)
Female	51.1 (207)
ITN coverage (N)	78.8 (319)
Educational level of parent/caregiver	
No formal	5.1 (19)
Primary	45.9 (170)
Secondary	39.7 (147)
Tertiary	9.2 (34)
Mosquito bed net use	
Yes	50.9 (206)
No	49.1(199)
Effective utilization of bed nets	
Yes	29.9 (121)
No	70.1 (284)
Integrity of mosquito bed net	
Good	71.6 (290)
Acceptable	6.9 (28)
Torn	0.2 (1)
Clinical	
Mean temperature (SD) in °C	36.71 (0.69)
Fever prevalence	7.9 (32)
Malaria parasite prevalence	46.7 (189)
Mean haemoglobin level (SD) in g/dl	10.95 (2.10)
Anaemia prevalence	54.6 (221)

### Malaria parasite prevalence and density

Overall, prevalence of malaria parasitaemia (MP) in the study population was 46.7% (189/405) and majority of the children had a low (74.6%, 141) followed by moderate (20.0%, 38) and high parasitaemia (5.4%, 10). As shown in Table 2, MP prevalence was similar between males (44.9%) and females (48.3%). A significant difference (P = 0.03) was observed with age, with the 5–9 years age group having the highest prevalence (54.6%). In addition, a significant difference (P = 0.02) was observed in the parasite density with respect to age; with the < 5 years age group having the highest geometric mean parasite density (GMPD)/µl of blood (537) and the least observed in the 10–14 years age group (319). Children who did not use ITNs as well as those whose ITNs were torn had a higher prevalence of MP than their respective counterparts although not statistically significant (Table 2).

**Table 2:** Malaria parasite prevalence and density with respect to sex, age, and ITN use

Parameter	No. examined	Prevalence (n)	P-value	Parasite density (parasites/µl of blood		P-value
				GMPD/µl	Range	
Gender						
Male	198	44.9 (89)	P = 0.50^c^	433	100–10,920	0.56^a^
Female	207	48.3 (100)		464	104–11,520	
Age group in years						
< 5	153	41.2 (63)	P = 0.03*^c^	538	104–11,520	0.02*^a^
5–9	163	54.6 (89)		456	100–10,920	
10–14	89	41.6 (37)		319	100–5740	
ITN ownership						
Yes	319	45.5(145)	P = 0.346^c^	474	100–11,520	0.65^a^
No	86	51.2 (44)		376	100–10,920	
ITN usage						
Yes	206	44.2 (91)	P = 0.306^c^	431	107–9300	0.63^a^
No	199	49.2 (98)		468	100–11,520	
Integrity of ITN						
Good	290	45.9 (133)	P = 0.438^c^	483	100–11,520	0.807^b^
Acceptable	28	39.3 (11)		416	138–1890	
Torn	1	100 (1)		170	170–170	

* Statistically significant at P < 0.05

^a^Difference in GMPD determined by Mann–Whitney

^b^Difference in GMPD determined by Krustal–Wallis

^c^Difference in proportions determined by Chi square

### Prevalence and severity of anaemia

The overall prevalence of anaemia was 54.6%. The prevalence of anaemia decreased significantly (P < 0.001) with an increase in age group. Contrarily, the youngest age group (< 5 years) had the highest prevalence of severe anaemia (5.2%), than those older (5–9 and 10–14 years) although the difference was not significant as shown in Table 3. Parasitaemic children and those who presented with fever had higher prevalence of anaemia (70.4% and 62.5%) than their respective negative counterparts. However, only the difference in prevalence by parasitaemia status was significant (P < 0.001). On the severity of anaemia, the proportion of parasitaemia positive children with severe to moderate anaemia was significantly lower (χ^2^ = 7.17, P = 0.03) than their negative counterparts. Similarly, the proportion of afebrile children with moderate and mild anaemia was significantly higher (χ^2^ = 10.65, P = 0.005) than in the febrile children as shown in Table 3

**Table 3:** Prevalence of anaemia and its severity as affected by age, malaria parasite and fever statuses

Variable	N	Anaemia	Chi square P-value	Anaemia severity				Chi square P-value
				N	Severe % (n)	Moderate % (n)	Mild % (n)	
Age group								
< 5	153	63.4 (97)	16.28 < 0.001***	97	5.2 (5)	55.7 (54)	39.2 (38)	4.47 0.35
5–9	163	55.8 (91)		91	2.2 (2)	56.0 (51)	41.8 (38)	
10–14	89	37.1 (33)		33	0 (0)	45.5 (15)	54.5 (18)	
Malaria parasite status								
Positive	189	70.4 (133)	35.70 < 0.001***	133	2.3 (3)	48.1 (64)	49.6 (66)	7.17 0.03*
Negative	216	40.7 (88)		88	4.5 (4)	63.6 (56)	31.8 (28)	
Fever status								
Febrile	32	62.5 (20)	0.88 0.36	31	12.9 (4)	48.4 (15)	38.7 (12)	10.65 0.005*
Afebrile	373	53.9 (201)		183	1.6 (3)	56.3 (103)	42.1 (77)	

* Statistically significant at P < 0.05

** Statistically significant at P < 0.01

*** Statistically significant at P < 0.001

### ITN ownership, utilisation rates and malariometric indices

**Fig. 2:**
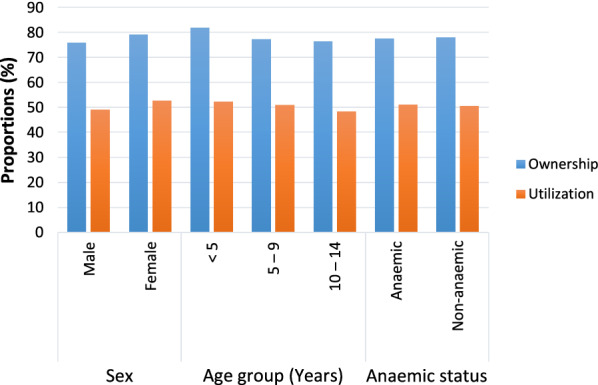
Ownership and utilization of ITN with respect to sex, age and anaemic status

As shown in Fig. 2, although not significant, the proportion of females with ITN (79.2%) and females who slept under bed nets (52.7%) were greater than the males while, with age the ownership and utilization was higher in the < 5 years age group than counterparts. Also, ownership and utilization of ITN among anaemic and non-anaemic children was comparable.

**Fig. 3:**
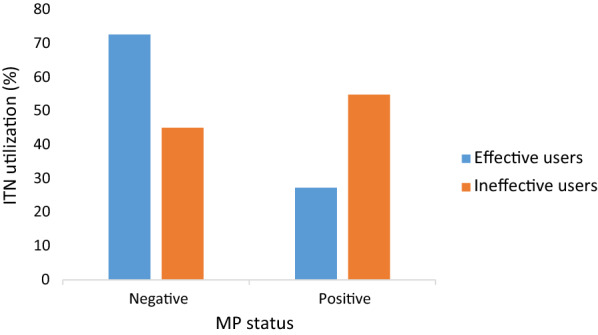
Effective utilization of ITN with respect to MP status

The proportion of ineffective users was significantly higher (χ^2^ = 26.08, P < 0.001) in children who were malaria parasite positive (54.9%) than negative (27.3%) as shown in Fig. 3.

**Fig. 4:**
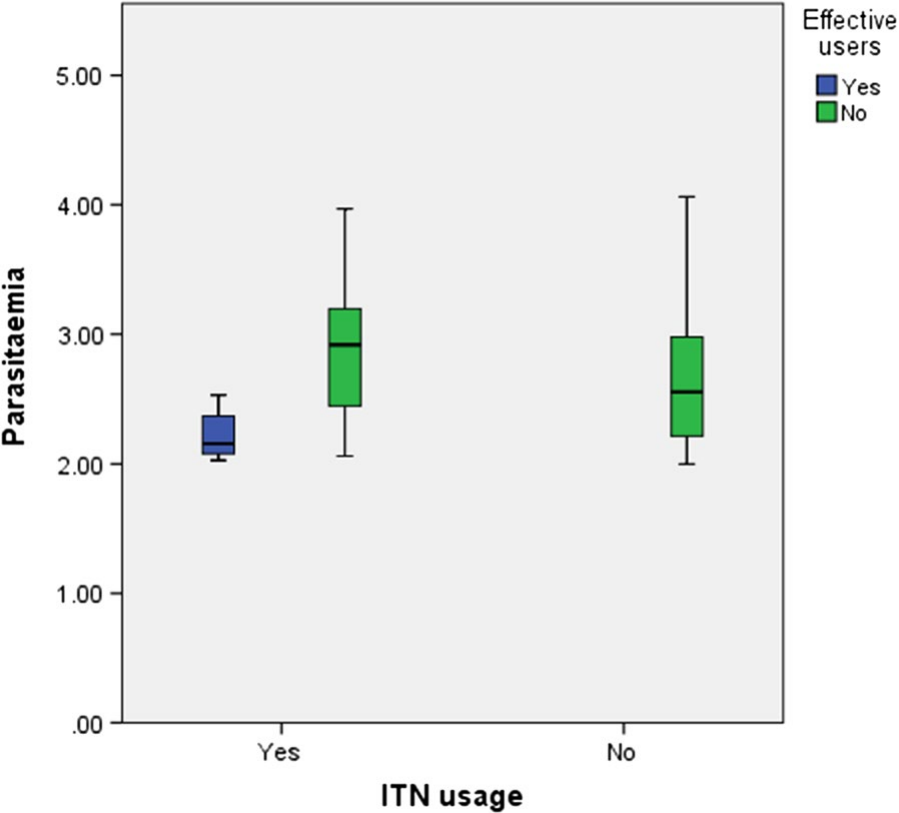
Mean parasitaemia density as affected by ITN usage

The mean parasite density was comparable between ITN users (2.63 parasite/µl of blood) and non ITN users (2.67 parasite/µl of blood). However, the mean parasite density was lower (t = − 5.138, P < 0.001) among children who effectively used ITN (2.24 parasite/µl of blood) compared to those who didn’t (2.74 parasite/µl of blood) as seen in Fig. 4.

### Knowledge and malaria parasite status

The overall appropriate knowledge on malaria was 40.7%. As shown in Table 4, there was a significant association (P < 0.001) between knowledge of malaria and parasitaemia status. The proportion of parasitaemia positive cases whose parents/caregivers had appropriate knowledge on malaria (30.7%) was significantly lower than their negative counterparts (49.5%). The level of appropriate knowledge of malaria decreased significantly (P = 0.001) with the level of education of parents/caregivers of the children (tertiary: 61.8% and no formal:21.1%) as shown in Table 4.

**Table 4:** Effects of clinical and demographic factors on malaria knowledge

Variable	N	Malaria knowledge % (n)		χ^2^ P
		Appropriate	Inappropriate	
Asexual malaria parasite status				
Negative	216	49.5 (107)	50.5 (109)	14.834 < 0.001***
Positive	189	30.7 (58)	69.3 (131)	
Age group of parents/caregivers				
< 30	143	34 (49)	66 (74)	5.85 0.05
31–40	172	43.2 (74)	56.8 (98)	
> 40	90	49.0 (41)	51.0 (46)	
Educational level of parents/caregivers				
No formal	19	21.1 (4)	78.9 (15)	13.26 < 0.001***
Primary	170	34.7 (59)	65.3 (111)	
Secondary	147	45.6 (67)	54.4 (80)	
Tertiary	34	61.8 (21)	38.2 (13)	

* Statistically significant at P < 0.05

*** Statistically significant at P < 0.001

### Malaria parasite, ITN, knowledge and haematological indices

The effect of malaria parasite on haematological indices is presented in Additional file 1 where, significantly lower Hb levels (P < 0.001), Hct (P < 0.001), and RBC counts (P < 0.001) were observed in malaria parasite positive children than negative. Also, children who used ITN had significant higher mean Hb levels (P = 0.032), RBC count (P = 0.009), MCHC (P = 0.039), RDW-CV (P = 0.036) and PLT count (P = 0.007) compared to those who did not use ITN as shown in Additional file 2.

Using a multilinear regression model with each haematological variable as the dependent variable, to examine the influence of age, gender, knowledge on malaria, ITN use, effective ITN use and parasitaemia status on each haematological variable; it was observed that age negatively correlated with WBC (P < 0.001) and positively correlated with Hb (P = 0.013), MCV (P < 0.001) and MCH (P < 0.001). Malaria knowledge negatively correlated with WBC (P = 0.005) but positively correlated with Hb levels (P < 0.001), RBC counts (P < 0.001), Hct (P < 0.001), MCV (P < 0.001) and MCH (P < 0.001). Similarly, ITN use positively correlated with WBC counts (P = 0.005) but negatively correlated with Hb levels (P = 0.004), RBC counts (P = 0.006), and MCH (P < 0.001). In addition, a significant negative correlation of effective ITN use with WBC (P = 0.006) was observed. Furthermore, MP negatively correlated with Hb levels (P = 0.004), RBC counts (P = 0.01), Hct (P = 0.04) and MCHC (P = 0.015) as shown in (Table 5).

**Table 5:** Multiple linear regression analyses examining the influence of independent variables on each haematologic measure

Haematological Parameter	Mean (SD)	Independent variable	β value	P value	Partial correlation	R^2^
WBC × 10^9^/l	7.9 (3.7)	Age	− 0.28	< 0.001***	− 1.39	0.14
		Sex	0.03	0.484	0.25	
		Knowledge	− 0.13	0.005**	− 1.02	
		ITN use	0.18	0.003**	1.37	
		Effective ITN use	− 0.17	0.006**	− 1.42	
		Malaria parasite	0.08	0.124	0.57	
Hb (g/dl)	11.0 (2.0)	Age	0.11	0.013*	2.95	0.26
		Sex	0.01	0.830	0.38	
		Knowledge	0.46	< 0.001***	19.3	
		ITN use	− 0.17	0.004**	− 6.80	
		Effective ITN use	0.09	0.143	3.86	
		Malaria parasite	− 0.15	0.004**	− 2.01	
RBC × 10^9^/l	5.0 (1.1)	Age	− 0.04	0.358	− 0.06	0.14
		Sex	0.03	0.481	0.07	
		Knowledge	0.32	< 0.001**	0.67	
		ITN use	− 0.17	0.006**	− 0.35	
		Effective ITN use	0.06	0.390	0.122	
		Malaria parasite	− 0.16	0.01**	− 0.126	
Hct (%)	36.1 (7.4)	Age	0.07	0.133	0.66	0.22
		Sex	0.03	0.495	0.45	
		Knowledge	0.42	< 0.001**	6.33	
		ITN use	− 0.11	0.07	− 1.56	
		Effective ITN use	0.03	0.585	0.53	
		Malaria parasite	− 1.63	0.04*	− 1.14	
MCV/(fl)	74.2 (8.7)	Age	0.16	0.001***	1.86	0.08
		Sex	− 0.06	0.220	− 1.03	
		Knowledge	0.17	< 0.001***	3.06	
		ITN use	0.09	0.169	1.52	
		Effective ITN use	− 0.05	0.473	− 0.89	
		Malaria parasite	− 0.05	0.310	− 0.91	
MCH/pg	22.6 (2.5)	Age	0.26	< 0.001***	0.86	0.13
		Sex	− 0.05	0.324	− 0.23	
		Knowledge	0.21	< 0.001***	1.05	
		ITN use	− 0.05	0.456	− 0.23	
		Effective ITN use	0.02	0.780	0.10	
		Malaria parasite	− 0.01	0.924	− 0.02	
MCHC (g/l)	305.9 (27.1)	Age	0.09	0.063	3.31	0.04
		Sex	− 0.01	0.923	− 0.26	
		Knowledge	0.08	0.105	4.51	
		ITN use	− 0.16	0.017*	− 8.45	
		Effective ITN use	0.10	0.153	5.68	
		Malaria parasite	0.13	0.015*	6.94	0.11
RDW-CV/%	15.9 (3.6)	Age	0.23	0.001***	1.03	
		Sex	0.09	0.049*	0.67	
		Knowledge	− 0.04	0.403	− 0.30	
		ITN use	− 0.29	0.001***	− 2.05	
		Effective ITN use	0.30	0.001***	2.32	
		Malaria parasite	0.10	0.060	0.68	
Plt/l	328.1 (141.1)	Age	o.15	0.003**	28.04	0.04
		Sex	0.02	0.765	4.15	
		Knowledge	0.02	0.733	4.94	
		ITN use	− 0.19	0.004**	− 52.25	
		Effective ITN use	0.09	0.194	26.81	
		Malaria parasite	0.04	0.932	1.27	

* Statistically significant at P < 0.05

** Statistically significant at P < 0.01

*** Statistically significant at P < 0.001

## Discussion

Insecticide-treated net use is a well-established malaria control intervention recommended in malaria endemic countries around the globe. Considerable progress with the use of interventions such as ITN and use of artemisinin-based combination therapy has been made in the past decade in reducing the burden of malaria in Africa, with Cameroon inclusive [4, 9]. The objective of this study was to determine the impact of ITN ownership and utilization as well as knowledge of malaria on malariometric and haematological indices in children in Batoke, a malaria *meso* endemic coastal town in the Mount Cameroon area.

The overall malaria parasitaemia of 46.7% in the population confirms earlier findings of the meso-to-hyper endemicity of malaria in the Mount Cameroon area [31, 32]. The observation is similar to the 47.5% reported by Sumbele et al*.* [31] among children less than 15 years in this lowland area and a 45.3% reported by Eyong et al. [32] in pupils between 4 and 16 years in other areas in the Mount Cameroon area. In addition, a similar prevalence (45.47%) was reported among school children in the Littoral Region, another coastal area of the country [33]. Worthy of note is that, this prevalence is lower than the 60.5% obtained by Ndamukong-Nyanga et al. [34] in a lowland study in the Mount Cameroon area. On the contrary, lower prevalence of 27.7% [35], 29.6% [15] and 32.9% [36] among a similar age group was reported in higher altitude areas, in the Mount Cameroon area and the Northern part of the country, respectively. This reveals that malaria remains a major cause of illness during childhood and the prevalence in this part of the country is not on a speedy decline. In addition, Batoke is at a lower altitude and several studies have reported that malaria prevalence is higher in lower altitude compared to their higher altitude counterparts [24, 25].

Interestingly, higher prevalence of the malaria parasite in children of the 5–9 years’ age group was consistent with the findings of Apinjoh et al. [15] within the same age group in the Mount Cameroon area. Also, higher prevalence among children aged 5–15 years compared to the under-five age group in this area has been reported by Ebai et al. [37]. Additionally, in another part of the Mount Cameroon area, Bate et al. [38] reported higher malaria prevalence among children aged 5–10 years when compared to their contemporaries. The epidemiological shift in malaria burden from the under-five age group to the 5–9 years age can be attributed to the playful and adventurous nature of children of this age group in the fields, which exposes them to the malaria vector*.* Another possible reason for this epidemiological shift in malaria prevalence from the less than five to the 5–9 years age group could be as a result of intensive malaria control including free LLINs distribution and ACT for less than five age group in all government health centres in Cameroon as decreed by the Head of state. Furthermore, the reduced malaria prevalence in the < 5 age group could be associated to parental guidance, bed sleeping time of the children of this age group and reinforcement on the proper use of these ITNs compared to the 5–9 age groups whose maternal care would have reduced and are more independent and less likely to use the ITNs.

Even though malaria parasite prevalence was lowest in the < 5 years age group, children in this group had the highest GMPD. The findings are in line with those of Achidi et al. [39]; Udoh et al*.* [40]; Bate et al. [38] carried out in Cameroon and Nigeria. Children under five years are more vulnerable to the disease in areas of high transmission [41] and it is often associated to the poorly developed immune system in this age group [42]. Observations from this study did not report any association between gender and *Plasmodium* infection, consistent with studies by Kwenti et al. [43] and Mbohou et al*.* [44] in other parts of the country.

The overall knowledge of malaria (i.e. mode of transmission, signs and symptoms, effects and control measures) in the study area is very low. Similar results were obtained by Birhanu et al. [45] in Ethiopia, whose study revealed that local understanding of malaria was unsatisfactory and very limited. Higher educational level was significantly associated with the knowledge of malaria. The findings are in congruence with those of Yaya et al*.* [46] who reported the likelihood of having accurate knowledge of malaria increased as the educational level increased. Additionally, Ndibuagu et al*.* [47] reported respondents with formal education had significantly better knowledge of malaria than those without formal education. Higher levels of education are generally associated with improved knowledge in relation to appropriate prevention and treatment strategies [3]. Consequently, this justifies the findings which revealed a higher prevalence of malaria parasite among children whose parents/caregivers were less educated. Therefore, they will put in less protective measures to reduce exposure thus preventing *Plasmodium* infection [48].

The prevalence of anaemia (54.6%) in children less than 15 years underscores the high burden of anaemia among the population in this area. However, this prevalence is lower compared to the 72.7% reported by Teh et al. [35] and the 62.0% and 75.3% reported by Sumbele et al. [4, 49] respectively, in the Mount Cameroon area. It is also lower than the national value of 62.5% [50]. It was however higher than the 44.8%, 49.6% and 44.7% reported by Njunda et al. [51], Bate et al. [38] and Tientche et al*.* [52] respectively in different locations in the Mount Cameroon area. The higher prevalence of anaemia in the population could be attributed to the burden of malaria in the population. A recent study [53] in 16 sub-Saharan African countries including Cameroon showed that anaemia is an important indicator for monitoring malaria burden.

Children of the < 5 years age group had a significantly higher occurrence of anaemia compared to their counterparts. The higher prevalence could be linked to the higher *Plasmodium* density observed in this age group. Similar findings were also reported by Sumbele et al*.* [49]. Udoh et al. [40] reported that, anaemia due to malaria is more severe in younger children in areas of intense transmission. Likewise, there was a negative correlation between Hb and malaria parasitaemia, further indicating that high parasite load causes more destruction of RBCs, consequently decreasing haemoglobin levels leading to anaemia.

The proportion of bed net possession was 78.8% among the children. The coverage rate was slightly lower than the WHO recommended 80% for acceptable protection. This ownership frequency is higher than the 63.5% obtained by Fokam et al. [5] in another part of Cameroon. Also, the ownership frequency was higher than that obtained in other countries, such as Angola (52.0%), and may be accounted for by the fact that during the free distribution campaign in Cameroon, all households were included, unlike in Angola where the distribution exercise was targeted [54]. With respect to utilization, 50.9% of the children had at least used a net the previous night, with an effective utilisation rate of 29.9%. These findings are in line with those of Fokam et al. [5] who reported that, there was a negative association between bed net ownership and utilization by households as bed net ownership was high and utilisation of these nets was low. Furthermore, Inungu et al. [55] in a study in the Democratic Republic of Congo reported lower utilization rates although ownership proportions were quite high (81.6%). These findings are not in conformity with those of Ntonifor and Veyufambom [6], where in a study carried out in the high western plateau part of the country observed that, most of the respondents that had nets usually sleep under them.

Observations from the study demonstrated significantly lower prevalence of malaria parasitaemia in children who effectively utilized the ITNs. This finding corroborates those of Ntonifor and Veyufambom, [6]. Insecticide-treated net is a highly effective means of preventing malaria vector transmission through a physical and chemical barrier, thereby reducing associated malaria morbidity and mortality, particularly in endemic areas [8]. Since transmission takes place mainly at night and, therefore, inside the house, the effective use of LLINs offers a degree of protection against the female *Anopheles* mosquito thus preventing malaria transmission [56].

This study confirms that haematological changes including RBC and its indices, are common complications encountered in *Plasmodium* parasite infection in children. Although malaria parasitaemia was not significantly associated with WBCs. The mean WBC counts in malaria infected and negative groups were normal and is in line with other studies [57]. Nevertheless, normal WBC counts are not enough to clearly indicate freedom from underlying disease. A differential analysis of the individual WBC counts which was a limitation of this study could have given a more precise blood picture. However, the finding contrasts those of Kimbi et al*.* [58] who indicated that as malaria parasitaemia rises in blood, the amount of WBC also rises. Moreover, Kotepui et al. [59] and Sumbele et al. [60], reported a lower amount of white blood cell count with increased parasitaemia.

Children who used ITN had significant higher mean Hb levels, RBC count, MCHC, RDW-CV and Plt count compared to those who did not use ITNs. Based on the results of the linear regression model, it was observed that ITN use had a significant influence on WBC, Hb, RBC, Hct, MCHC, RDW-CV and Plt. The significantly higher Hb, RBC, MCHC, RDW-CV and Plt count in ITN users than non-users, may be as a result of the protective efficacy of the ITNs that prevents the ITN users from the bite of the *Anopheles* vector thereby preventing them from contracting the *Plasmodium* infection, which has been proven in several studies [58, 61, 62] to reduce the haematological parameters of children. This finding corroborates with results of Maina et al. [61] and Kotepui et al*.* [59] who reported that; red blood cell counts, haemoglobin and platelets counts were significantly lower in malaria positive children than negative children. The significantly lower RBCs in malaria positive children than negative children could be associated with haemolysis, cytoadherance, rosetting, clumping [63]. Furthermore, significantly lower levels of haemoglobin in malaria positive than negative children can be attributed to the digestion of haemoglobin by erythrocytic stages of the malaria parasite.

The age of the children had a significant influence on MCV, MCH, RDW-CV and Plt counts. Likewise, the gender had a significant influence on the WBC counts, Hb and RDW-CV of the children. The results are in line with those of Onwurah et al*.* [64] who reported that age and gender have an effect on the haematological parameters. This study suggests that changes in haematological parameters accompanied with age and gender may depend on the population and geographical area studied [65] and, therefore, reference values validated for one country should not be assumed for application in population from other countries [66].

As a limitation, the study did not explore other personal and behavioural factors such as attitude, beliefs and perception regarding the role of ITN and health care-seeking which have been reported to influence behaviours.

## Conclusions

Findings of this study revealed that malaria remains prevalent and is a major cause of morbidity in children less than 15 years of age in Batoke–Limbe, Mount Cameroon area. Despite the efforts put in place by the government of Cameroon to scale up ITN distribution so that universal coverage can be attained, coverage remains lower than the 80% recommended by the WHO. Effective utilization of ITN is low and is significantly associated with a decrease in parasite prevalence hence nation-wide campaigns to educate the population on proper use of ITN should be carried out. The overall knowledge of malaria in the study area is very low and improved malaria knowledge is associated with higher level of education hence, the higher prevalence of malaria parasite among children whose parents/ caregivers were less educated. Furthermore, ITN users had a higher Hb, RBC, MCHC, RDW-CV and Plt count than non-users, demonstrating a protective efficacy of the ITNs by not only preventing *Plasmodium* transmission but reducing morbidity as well.

## Supplementary Information

**Additional file 1. **Mean haematological indices as affected by malaria parasite status.

**Additional file 2. **Mean haematological indices as affected by ITNs use.

## Data Availability

All datasets on which the conclusions of the research rely are presented in this paper. However, data is available from the corresponding author on reasonable request.

## Abbreviations

ITN: Insecticide-treated net
LLIN: Long-lasting insecticidal net
WBC: White blood cell
RBC counts: Red blood cell
Hb level: Haemoglobin
Hct: Haematocrit
MCV: Mean corpuscular volume
MCH: Mean corpuscular haemoglobin
MCHC: Mean corpuscular haemoglobin concentration
Plt: Platelet counts
