# Supplementary material for: Insecticide-treated net ownership, utilization and knowledge of malaria in children residing in Batoke–Limbe, Mount Cameroon area: effect on malariometric and haematological indices
Source: Malar J. 2021 Jul 29;20:333. doi: 10.1186/s12936-021-03860-6 (PMC8320188; doi:10.1186/s12936-021-03860-6)
Supplement: Supplementary file 1 — Additional file 1. Mean haematological indices as affected by malaria parasite status. [file 12936_2021_3860_MOESM1_ESM.docx]

**Additional file 1. Mean haematological indices as affected by malaria parasite status**

| Parameter | Malaria parasite status | Number examined | Mean ± SD | Range | Level of significance |
| --- | --- | --- | --- | --- | --- |
| WBC × 10^9^/ L | Negative | 216 | 7.3 ± 2.6 | 2.0 – 16.0 | t = 1.952  P = 0.052 |
|  | Positive | 189 | 7.8 ± 2.5 | 3.0 – 19.0 |  |
| Hb (g/dl) | Negative | 216 | 11.2 ± 2.0 | 5.9 – 17.0 | t = -2.908  P < 0.001* |
|  | Positive | 189 | 10.6 ± 1.8 | 6.0 – 15.7 |  |
| RBC× 10^9^/ L | Negative | 216 | 5.1 ± 1.1 | 2.0 – 9.0 | t = -2.908  P < 0.001* |
|  | Positive | 189 | 4.8 ± 1.0 | 3.0 – 9.0 |  |
| Hct (%) | Negative | 216 | 36.8 ± 6.3 | 20.0 – 59.0 | t = -2.832  P < 0.001* |
|  | Positive | 189 | 34.9 ± 7.0 | 18.0 – 57.0 |  |
| MCV/ (fl) | Negative | 216 | 74.5 ± 7.4 | 53.0 – 10.4 | t = -1.141  P = 0.254 |
|  | Positive | 189 | 73.6 ± 8.9 | 50.0 – 104.0 |  |
| MCH/pg | Negative | 216 | 22.7 ± 2.7 | 15.0 – 33.0 | t = -0.771  P = 0.441 |
|  | Positive | 189 | 22.5 ± 2.2 | 17.0 – 29.0 |  |
| MCHC (g/L) | Negative | 216 | 303.4 ± 24.9 | 213.0 – 355.0 | t = 1.984  P = 0.048* |
|  | Positive | 189 | 308.7 ± 29.2 | 224.0 – 346.0 |  |
| RDW-CV/% | Negative | 216 | 16.0 ± 4.0 | 10.0 – 42.0 | t = -0.705  P = 0.482 |
|  | Positive | 189 | 15.7 ± 3.0 | 10.0 – 29.0 |  |
| Plt/L | Negative | 216 | 328.2 ± 147.4 | 36.0 – 950 | t = 0.066  P = 0.947 |
|  | Positive | 189 | 327.3 ± 147.4 | 47.0 – 876 |  |

*Statistically significant at P < 0.05
