# Supplementary material for: Insecticide-treated net ownership, utilization and knowledge of malaria in children residing in Batoke–Limbe, Mount Cameroon area: effect on malariometric and haematological indices
Source: Malar J. 2021 Jul 29;20:333. doi: 10.1186/s12936-021-03860-6 (PMC8320188; doi:10.1186/s12936-021-03860-6)
Supplement: Supplementary file 2 — Additional file 2. Mean haematological indices as affected by ITNs use. [file 12936_2021_3860_MOESM2_ESM.docx]

Additional file 2. Mean haematological indices as affected by ITNs use

| Parameter | ITN Use | Number examined | Mean ± SD | Range | Level of significance |
| --- | --- | --- | --- | --- | --- |
| WBC × 10^9^/ L | Yes | 206 | 8.1 ± 4.0 | 3.2 – 33.0 | t = 1.460  P = 0.145 |
|  | No | 199 | 7.6 ± 3.5 | 2.2 – 31.4 |  |
| Hb (g/dL) | Yes | 206 | 11.1 ± 2.4 | 5.6 – 23.6 | t = 2.156  P = 0.032* |
|  | No | 199 | 10.8 ± 1.6 | 5.9 – 17.6 |  |
| RBC × 10^9^/ L | Yes | 206 | 5.1 ± 1.2 | 2.5 – 8.9 | t = 2.612  P < 0.001* |
|  | No | 199 | 4.8 ± 0.8 | 2.4 – 7.9 |  |
| Hct (%) | Yes | 206 | 36.7 ± 8.7 | 18.0 – 74.0 | t = -1.594  P = 0.112 |
|  | No | 199 | 35.6 ± 5.7 | 20.0 – 59.0 |  |
| MCV/ (fl) | Yes | 206 | 74.7 ± 9.2 | 51.1 – 107.7 | t = 1.191  P = 0.234 |
|  | No | 199 | 73.7 ± 8.0 | 49.7 – 111.2 |  |
| MCH/pg | Yes | 206 | 22.5 ± 2.4 | 15.3 – 33.2 | t = 0.764  P = 0.445 |
|  | No | 199 | 22.7 ± 2.6 | 15.6 – 43.3 |  |
| MCHC g/L | Yes | 206 | 308.7 ± 27.1 | 225.0 – 400.0 | t = 2.069  P =0.039* |
|  | No | 199 | 303.2 ± 26.8 | 213.0 – 403.0 |  |
| RDW-CV/% | Yes | 206 | 16.2 ± 4.1 | 9.9 – 42.1 | t = 2.099  P =0.036* |
|  | No | 199 | 15.5 ± 2.9 | 9.8 – 32.7 |  |
| Plt/L | Yes | 206 | 346.7 ± 147.3 | 36.0 – 950.0 | t = 2.708  P < 0.001* |
|  | No | 199 | 338.2 ± 147.4 | 36.0 – 950.0 |  |

*statistically significant
